# Predictors of treatment failures of *plasmodium falciparum* malaria in Vietnam: a 4-year single‐centre retrospective study

**DOI:** 10.1186/s12936-021-03720-3

**Published:** 2021-04-29

**Authors:** Minh Cuong Duong, Oanh Kieu Nguyet Pham, Phong Thanh Nguyen, Van Vinh Chau Nguyen, Phu Hoan Nguyen

**Affiliations:** 1 School of Population Health, University of New South Wales, Sydney, Australia; 2 Hospital for Tropical Diseases, Ho Chi Minh City, Vietnam; 3 Oxford University Clinical Research Unit (OUCRU), Ho Chi Minh City, Vietnam; 4 Medical School, Vietnam National University of Ho Chi Minh City, Ho Chi Minh City, Vietnam

**Keywords:** *Plasmodium falciparum*, Severe malaria, Early treatment failure, Late treatment failure, Vietnam

## Abstract

**Background:**

Drug-resistant falciparum malaria is an increasing public health burden. This study examined the magnitude of *Plasmodium falciparum* infection and the patterns and predictors of treatment failure in Vietnam.

**Methods:**

Medical records of all 443 patients with malaria infection admitted to the Hospital for Tropical Diseases between January 2015 and December 2018 were used to extract information on demographics, risk factors, symptoms, laboratory tests, treatment, and outcome.

**Results:**

More than half (59.8%, 265/443, CI 55.1–64.4%) of patients acquired *Plasmodium falciparum* infection of whom 21.9% (58/265, CI 17.1–27.4%) had severe malaria, while 7.2% (19/265, CI 4.6–10.9%) and 19.2% (51/265, CI 14.7–24.5%) developed early treatment failure (ETF) and late treatment failure (LTF) respectively. Among 58 patients with severe malaria, 14 (24.1%) acquired infection in regions where artemisinin resistance has been documented including Binh Phuoc (11 patients), Dak Nong (2 patients) and Gia Lai (1 patient). Under treatment with intravenous artesunate, the median (IQR) parasite half-life of 11 patients coming from Binh Phuoc was 3 h (2.3 to 8.3 h), two patients coming from Dak Nong was 2.8 and 5.7 h, and a patient coming from Gia Lai was 6.5 h. Most patients (98.5%, 261/265) recovered completely. Four patients with severe malaria died. Severe malaria was statistically associated with receiving treatment at previous hospitals (P < 0.001), hepatomegaly (P < 0.001) and number of inpatient days (P < 0.001). Having severe malaria was a predictor of ETF (AOR 6.96, CI 2.55–19.02, P < 0.001). No predictor of LTF was identified.

**Conclusions:**

*Plasmodium falciparum* remains the prevalent malaria parasite. Despite low mortality rate, severe malaria is not rare and is a significant predictor of ETF. To reduce the risk for ETF, studies are needed to examine the effectiveness of combination therapy including parenteral artesunate and a parenteral partner drug for severe malaria. The study alerts the possibility of drug-resistant malaria in Africa and other areas in Vietnam, which are known as non-endemic areas of anti-malarial drug resistance. A more comprehensive study using molecular technique in these regions is required to completely understand the magnitude of drug-resistant malaria and to design appropriate control strategies.

## Background

Malaria is a mosquito-transmitted infection that affects 219 million people and causes 435 thousand deaths worldwide [1]. Among the 5 *Plasmodium* species causing malaria in humans, *Plasmodium falciparum* is responsible for the most severe forms of malaria [2]. In Vietnam, *P. falciparum* is the most prevalent malaria parasite followed by *Plasmodium vivax* [3]. Although the prevalence of malaria infection decreased in the last two decades, anti-malarial drug resistance has been emerging in Vietnam [4, 5]. Dihydroartemisinin (DHA), which is an artemisinin derivative, has been used for treating malaria infection in Vietnam for more than 20 years [6]. In line with the World Health Organization (WHO) recommendations [7], artemisinin-based combination therapy (ACT), including DHA and piperaquine (PPQ) has been a mandatory first choice treatment of uncomplicated *P. falciparum* infection since 2007 [8]. However, molecular markers for artemisinin resistance were detected in Cambodia in 2009 and subsequently spread rapidly to other countries in the Greater Mekong Subregion (GMS) including Vietnam [9, 10]. Moreover, resistance to PPQ has also been confirmed [11]. Recently artesunate-pyronaridine has been recommended for uncomplicated *P. falciparum* infection in Dak Nong and Binh Phuoc which are the two provinces mostly affected by DHA-PPQ resistance [12].

Diagnostic tests used to identify anti-malarial drug resistance such as molecular assays are not always available, especially in low-resource countries [13]. Therefore, monitoring response to treatment, mainly parasite clearance time and fever clearance time are recommended in the clinical management of patients with malaria and the surveillance of anti-malarial drug resistance [13]. The resistance to DHA-PPQ combination therapy causes delayed parasite clearance time [14]. The susceptibility of *P. falciparum* to DHA-PPQ combination therapy in southern Vietnam has declined rapidly with the increase in the proportion of patients with parasite clearance time of more than 72 h (from 38% to 2012 to 57% in 2015) [5]. To strengthen the current strategies to control the spread and impact of drug*-*resistant *P*. *falciparum* in Vietnam and other countries in the GMS, it is important to know the current burden of *P. falciparum* infection and the effectiveness of anti-malarial drugs in Vietnam. The Hospital for Tropical Diseases (HTD) in Ho Chi Minh City is a tertiary hospital which receives patients with malaria from Central Highland and southern Vietnam including the Vietnam–Cambodia border area. The presenting study was conducted at this hospital to examine the magnitude of *P. falciparum* infection, severe malaria and the response to anti-malarial treatment including the patterns of and predictors for treatment failure.

## Methods

### Design of the study

Medical records of all patients with malaria admitted to the HTD between January 2015 and December 2018 were retrieved for review. Information derived from medical records of patients with *P. falciparum* infection was extracted and included demographics (age, gender, job, and residential address), risk factors (blood transfusion, injecting drug use (IDU), travelling to malaria endemic areas domestically and internationally within 14 days before the onset of illness [15]), current health conditions (pregnancy, end stage renal disease, cirrhosis, and HIV infection), malaria disease and treatment at previous hospitals and HTD. It is noted that HTD is the tertiary teaching hospital in southern Vietnam for infectious diseases including malaria. Therefore, as required by the HTD policy, this information must be obtained correctly and entered into the medical record. The data collection was performed by two authors (OKNP and PTN) who are infectious disease specialists and provide malaria treatment to patients at the HTD. Any discrepancies derived from the data collection process were cross-checked by these authors and the other local research assistants who are also clinical doctors until consensus was obtained. It is noted that if patients have any risk factor for HIV infection, they will be consulted to undertake HIV testing in accordance with the HTD guideline. Information on malaria disease and treatment included admission time, number of days of illness at the time of admission, symptoms and signs (fever, anaemia, splenomegaly, and hepatomegaly), laboratory tests (malaria microscopy, parasite counts, and aminotransaminases (AST and ALT)), anti-malarial and other supportive treatments, response to treatment (number of inpatient days, fever clearance time, parasite clearance time, and early (ETF) and late treatment failure (LTF)) [16], outcomes (recovery and death), and having had malaria previously.

All laboratory tests including microscopy were performed at the HTD and in line with the national laboratory performance standards. According to the HTD policy, all cases were diagnosed using microscopy. Once the diagnosis is confirmed, patients must be screened for all clinical and laboratory signs of severe malaria. Blood smear for the presence of parasites and parasite density is performed at an interval of six (severe malaria) or 12 h (uncomplicated malaria) until blood smear shows a negative result for at least two times. Based on the course of the disease, *P. falciparum* infection was classified into different types of severe malaria in accordance with the WHO guidelines for the treatment of malaria and included shock, acute kidney failure, impaired consciousness, jaundice, anaemia, haemoglobinuria, acidosis, hyperparasitaemia, prostration, convulsion, hypoglycaemia and bleeding [7]. Regarding shock, the WHO guidelines were updated during the period when the presenting study was conducted. In line with the local requirement, shock definition was updated accordingly based on the update of World Health Organization (WHO) guidelines. In detail, for those who admitted to the HTD before September 2016, shock was defined as systolic blood pressure < 50 mmHg in children or < 80 mmHg in adults, with evidence of impaired perfusion (cool peripheries or prolonged capillary refill) [17]. Since September 2016, shock has been defined as systolic blood pressure < 90 mmHg in adults or a drop of 20 mmHg in systolic pressure by age in children, with evidence of impaired perfusion [18]. ETF and LTF were defined according to the WHO recommendations [16] and the distribution of these treatment failures was also examined. However, it is documented that in an area of artemisinin resistance, patients with prolonged parasitaemia including those having parasitaemia on day 3 with or without axillary temperature ≥ 37.5 °C respond well to ACT treatment [14, 19]. Therefore, in the presenting study, ETF did not include these people provided that the study was conducted in an area of artemisinin resistance. ETF included (i) danger signs or severe malaria on day 1, 2 or 3, in the presence of parasitaemia; or (ii) parasitaemia on day 2 higher than on day 0, irrespective of axillary temperature; and (iii) parasitaemia on day 3 ≥ 25% of count on day 0. ETF and LTF are not routinely assessed for all patients admitted to the HTD.

The presence of ETF among study participants was examined based on the clinical symptoms and laboratory tests. Regarding LTF, although study participants were not followed up on day 28 and day 42 after anti-malarial treatment due to the nature of a retrospective cohort study, information on previous malaria infection within 28 days was documented in the medical record as required by the HTD policy. Therefore, patients with LTF in the presenting study were those who met the WHO definition of LTF within 28 days before admission. In detail, LTF includes (i) danger signs or severe malaria in the presence of parasitaemia on any day between day 4 and day 28 in patients who did not previously meet any of the criteria of early treatment failure and (ii) presence of parasitaemia on any day between day 4 and day 28 (day 42) with axillary temperature ≥ 37.5 °C in patients who did not previously meet any of the criteria of early treatment failure, or (iii) presence of parasitaemia on any day between day 7 and day 28 with axillary temperature < 37.5 °C in patients who did not previously meet any of the criteria of early treatment failure or late clinical failure. Before September 2016, uncomplicated *P. falciparum* infection was treated with a three-day DHA-PPQ age-based dosing therapy. Severe malaria was treated with intravenous artesunate 2.4 mg/kg on admission, at 12 h, and then every 24 h until oral therapy can be given [17]. Since September 2016, although the anti-malarial agents remained the same, weight-based dosing has been used for uncomplicated malaria. Regarding severe malaria, the dosage of intravenous artesunate remained unchanged but the maximum duration of artesunate treatment has been set at seven days [18]. Given these changes in malaria treatment [18, 20], the changes in the magnitude of malaria and severe malaria before and after 2017 were also examined. According to the local guidelines, in order to be discharged from the hospital, patients must meet four criteria including receiving a full course of anti-malarial therapy, no fever, no severe malaria symptoms, and blood smear negative for parasites for at least two times. Therefore, patients who did not meet all these criteria were followed up in our hospital until discharge. The study protocol was approved by the HTD’s Ethics Committee (approval number 65/QD-BVBND) and the Human Research Ethics Committee at UNSW Australia (approval number HC180340).

### Statistical analysis

Data were stored and analysed using SPSS version 22 (IBM). For comparison purposes, a calculation of 95% confidence interval (CI) for the prevalence of *P. falciparum* infection was performed based on the number of subjects with malaria infection and the point estimate of the prevalence of *P. falciparum* infection. Similar calculations were performed for the prevalence of severe malaria, ETF and LTF. Chi-square, chi-square for trend and Fisher’s Exact tests were used to compare categorical data. Student’s t-test was used to compare continuous data. Multinomial logistic regression models were developed to test predictors of ETF and LTF. Independent variables including age, severe malaria, and acquiring malaria before 2017 were entered into the ETF model. Similarly, age, severe malaria, and patients with parasitaemia 72 h of treatment were entered into the LTF model. The significant level was set at *P* ≤ 0.05.

## Results

### Demographic characteristics of study participants

**Fig. 1:**
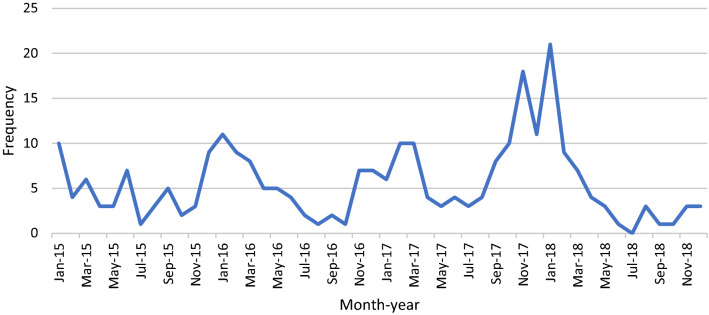
Frequency distribution of *P. falciparum* infected cases between January 2015 and December 2018

There were 443 malaria infected patients admitted to our hospital between January 2015 and December 2018. Of these patients, *P. falciparum* malaria accounted for 59.8% (265/443, CI 55.1–64.4%). *Plasmodium vivax* and *Plasmodium malariae* accounted for 35.7% (158/443, CI 31.4 − 40.2%) and 3.6% (16/443, CI 2.2 − 5.8%), respectively, while mixed *P. vivax–P. falciparum* infections accounted for 0.9% (4/443, CI 0.4–2.3%). Among 265 *P. falciparum* infected patients, nearly half (44.5%, 118/265) of them acquired infection before 2017 and most of them (83.4%) were male with the mean age of 35.3 ± 13.1 years (Table 1). More than half (56.3%) of patients had jobs related to the forest or worked in malaria endemic areas, including mountain farmers (29.1%), forest workers (8.7%), forest ranger (1.9%), healthcare worker working in the forest (0.4%) and working in Cambodia and Africa (16.2%). Having previous blood transfusions was documented in one patient and each of IDU and pregnancy was noted in the other two patients. No one had HIV infection. Nearly half (44.9%, 119/265) of study participants lived in malaria endemic areas and 36.6% (97/265) travelled to endemic areas within 14 days prior to the onset of disease. The number of cases was peaked during the period between November and March annually (Fig. 1).

**Table 1:** Demographic characteristics of 265 *P. falciparum* infected patients receiving treatment at the Hospital for Tropical Diseases between 2015 and 2018

Characteristics	Summary statistics^a^
Male	83.4 (221)
Age (years)	35.3 ± 13.1
Jobs	
Forest ranger	1.9 (5)
Mountain farmer	29.1 (77)
Forest worker	8.7 (23)
Healthcare worker working in the forest	0.4 (1)
Working in Cambodia	6.8 (18)
Working in Africa	9.4 (25)
Others*	43.7 (116)
Acquiring malaria before 2017	44.5 (118)
Concurrent health condition and lifestyle factors	
Blood transfusion	0.4 (1)
IDU	0.8 (2)
Pregnancy	0.8 (2)
Living in or travelling to malaria endemic areas 14 days prior to the onset of disease	
Living in endemic areas	44.9 (119)
Travelling to endemic areas	36.6 (97)
Unknown	18.5 (49)

^a^%(N) for categorical variables and Mean ± SD for continuous variables

*Others include office workers, teachers, and retailers

### Clinical manifestations of *P. falciparum* infected patients

Almost all patients (99.6%, 264/265) had fever on admission, while only 0.8% had anaemia, 3% had splenomegaly, and 10.2% had hepatomegaly (Table 2). There were 84 (31.7%) patients receiving treatment at previous hospitals. Among these patients, three received oral quinine at previous hospitals, of whom two used quinine for two days and one used seven days. Parasitaemia still persisted in all of them at the time of admission. At the HTD, two cases taking two-day quinine therapy were treated with a three-day DHA-PPQ therapy and a case taking seven-day quinine therapy was treated with a four-day intravenous artesunate followed by three-day DHA-PPQ therapy, of whom the latter developed ETF. All of them completely recovered in the end. None of study participants was treated with quinine at the HTD. A quarter (25.3%, 67/265) of patients had developed illness for more than seven days at the time of admission. One third of study participants had AST (31.3%, 83/265) and ALT (30.2%, 80/265) > 40 U/L, of whom the mean AST and ALT levels were 99.6 ± 98.8 U/L and 98.1 ± 60.2 U/L, respectively.

**Table 2:** Clinical patterns of *P. falciparum* infection among 265 infected patients receiving treatment at the Hospital for Tropical Diseases between 2015 and 2018

Characteristics	Summary statistics^a^
Signs and symptoms	
Fever on admission	99.6 (264)
Anaemia	0.8 (2)
Splenomegaly	3 (8)
Hepatomegaly	10.2 (27)
Severe malaria	21.9 (58)
Manifestations of severe malaria (n = 58)	
Single manifestations	77.6 (45)
Shock	8.6 (5)
Acute kidney failure	6.9 (4)
Impaired consciousness*	20.7 (12)
Jaundice	24.1 (14)
Anaemia	6.9 (4)
Haemoglobinuria	1.7 (1)
Acidosis	1.7 (1)
Hyperparasitaemia	6.9 (4)
Prostration	0
Convulsions	0
Hypoglycaemia	0
Bleeding	0
Mixed manifestations (i.e., patients had more than one severe manifestation)	22.4 (13)
Receiving treatment at previous hospital	31.7 (84)
Diagnosis at previous hospital (n = 84)	
Malaria	78.6 (66)
Others (i.e., dengue infection and septicaemia)	21.4 (18)
Number of days of illness > 7 days at the time of admission (days)	25.3 (67)
Number of days of illness at the time of admission (days)	6.6 ± 5.1
AST > 40 U/L	31.3 (83)
Highest AST recorded (U/L) (n = 83)	99.6 ± 98.8
ALT > 40 U/L	30.2 (80)
Highest ALT recorded (U/L) (n = 80)	98.1 ± 60.2

^a^%(N) for categorical variables and Mean ± SD for continuous variables

* Glasgow coma score < 11 in adults. There were 19 children patients in this study and none of them had impaired consciousness

Fifty-eight patients (21.9%, 58/265, CI 17.1–27.4%) had severe malaria, of whom single manifestation accounted for 77.6% (45/58, CI 64.7–87.5%) and 4 patients died. Eleven patients were from Binh Phuoc, 10 from Africa, 9 from Cambodia, two from Dak Nong, and one from each of the provinces including Binh Thuan, Dong Nai and Gia Lai. The remaining 23 patients had an unknown history of coming from or travelling to malaria endemic areas. Among 58 patients with severe malaria, 82.8% (48/58) were male and the mean age was 37.38 ± 14.17 years old. One patient (1.7%) had a history of injecting drug use. The mean number of days of illness at the time of admission was 6.9 ± 4.4. Fifteen patients (25.9%) had a number of days of illness > 7 days at the time of admission. The most common manifestations included jaundice (24.1%, 14/58), impaired consciousness (20.7%, 12/58), and shock (8.6%, 5/58). All five patients developing shock were adults, of whom three admitted to our hospital before September 2016 and all had a systolic blood pressure of ≤ 80 mmHg at the time of shock. Just under half (24/58, 46.2%) developed splenomegaly, hepatomegaly, or both, 13 (22.4%) required mechanical ventilation, 8 (13.8%) required haemodialysis, 12 (20.7%) received red blood cell transfusion. ETF and LTF developed in 12 (20.7%) and 8 (13.8%) patients with severe malaria, respectively. In the 58 patients presenting with severe falciparum malaria, 14 (24.1%) patients acquired malaria infection in regions of Vietnam where artemisinin resistance has been documented including Binh Phuoc (11 patients), Dak Nong (2 patients) and Gia Lai (1 patient). Under treatment with intravenous artesunate, the median (IQR) parasite half-life of 11 patients coming from Binh Phuoc was 3 h (2.3 to 8.3 h), while the parasite half-life of two patients coming from Dak Nong was 2.8 and 5.7 h and the parasite half-life of a patient coming from Gia Lai was 6.5 h. One (7.1%, 1/14) had a fatal outcome and was from Binh Phuoc. None of them required rescue treatment with parenteral quinine.

Having fever and parasitaemia after 3 days of anti-malarial treatment were documented in 18 (31%) and 11 (19%) patients with severe malaria, respectively. Among 4 patients with severe malaria died, one patient was a 41-year-old male with an unknown history of travelling to malaria endemic areas who presented with impaired consciousness, acute kidney failure, and jaundice. Another patient was a 16-year-old female coming from Binh Phuoc with impaired consciousness, anaemia, and jaundice. The remaining two patients were those coming from or travelling to Africa with disease manifestations outlined the section listing the characteristics of patients coming from or travelling to Africa below. The mean number of inpatient days was 7.9 ± 4.3. Eleven patients developed hospital acquired infections which were described in the section listing the characteristics of patients with hospital acquired infections below. Based on the availability of the parasite count results among 58 patients with severe malaria, the mean parasite density was documented at 183,287 ± 321,304 at the start of ACT among 58 patients, 153,095 ± 205,081 at the 6th hour among 45 patients, 88,029 ± 142,219 at the 12nd hour among 58 patients, 46,226 ± 91,477 at the 24th hour among 46 patients, 2552 ± 2272 at the 36th hour among 46 patients, and 1040 ± 1,180 at the 48th hour among 35 patients. The mean parasite half-life (50% reduction in parasitaemia during the linear phase of the log-parasitaemia over time) was 4.7 ± 2.1 h.

### Treatment, response to treatment, and outcome of *P. falciparum* infected patients

In addition to anti-malarial treatment, 5.7% (15/265) of patients received red blood cell transfusion, 4.9% (13/265) received mechanical ventilation, and 3% (8/265) undertook haemodialysis (Table 3). Hospital acquired infection was developed in 4.2% (11/265) of patients, of whom six developed pneumonia, four developed urinary tract infection, and one developed septicemia. Comorbidities were found in three patients including one with hypertension and type 2 diabetes, one with chronic obstructive pulmonary disease, and one with immune thrombocytopenia. Of these 11 patients with hospital acquired infections, 10 patients developed severe malaria including one with shock, two with acute kidney failure, three with impaired consciousness, two with jaundice, and two with mixed manifestations. One patient died. Extended-Spectrum Beta-Lactamase (*ESBL*)-producing *Escherichia coli* (two patients), *Klebsiella* pneumoniae (one patient), and *Pseudomonas aeruginosa* (two patients) were detected. The infectious agent was not detected in the remaining six patients.

**Table 3:** Treatment and outcome of 265 *P. falciparum* infected patients receiving treatment at the Hospital for Tropical Diseases between 2015 and 2018

Characteristics	Summary statistics^a^
Mechanical ventilation	4.9 (13)
Haemodialysis	3 (8)
Red blood cell transfusion	5.7 (15)
Hospital acquired infection (HAI)	4.2 (11)
Patients with fever 3 days of treatment	13.2 (35)
Fever clearance times (days)	2.4 ± 1.5
Patients with parasitaemia 72 h of treatment	15.1 (40)
Parasite clearance times (hours)	53 ± 30.8
Early treatment failure	7.2 (19)
Distribution of early treatment failure	
Vietnam	
Binh Phuoc and Central Highland (n = 148)	4.7 (7)
Other areas (n = 62)	12.9 (8)
Cambodia (n = 26)	7.7 (2)
Africa (n = 29)	6.9 (2)
Patients with parasitaemia on day 3 with axillary temperature ≥ 37.5 °C	3 (8)
Late treatment failure	19.2 (51)
Distribution of late treatment failure	
Vietnam	
Binh Phuoc and Central Highland (n = 148)	16.2 (24)
Other areas (n = 62)	22.6 (14)
Cambodia (n = 26)	19.2 (5)
Africa (n = 29)	27.6 (8)
Number of inpatient days	5.7 ± 3.5
ICU admission	10.9 (29)
Response to treatment	
Recovery	98.5 (261)
Death	1.5 (4)

^a^%(N) for categorical variables and Mean ± SD for continuous variables

Thirty-five (13.2%) and 40 (15.1%) patients had fever and parasitaemia after 3 days of anti-malarial treatment, respectively. The mean fever clearance time was 2.4 ± 1.5 days and the mean parasite clearance time was 53 ± 30.8 h. ETF and LTF were documented in 19 (7.2%, CI 4.6−10.9%) and 51 (19.2%, CI 14.7−24.5%) patients, respectively. ETF accounted for 4.7% (CI 2.3−9.4%) of patients coming from or travelling to Binh Phuoc and Central Highland, 12.9% (CI 6.7−23.5%) from other areas in Vietnam, 7.7% (CI 2.1−24.1%) from Cambodia and 6.9% (CI 1.9−22.0%) from Africa. Similarly, the proportion of LTF was 16.2% (CI 11.2 − 23.0%) among patients coming from or travelling to Binh Phuoc and Central Highland, 22.5% (CI 14.0−34.4%) from other areas in Vietnam, 19.3% (CI 8.5−37.9%) from Cambodia and 27.6% (CI 14.7−45.7%) from Africa. The proportion of patients with parasitaemia on day 3 with axillary temperature ≥ 37.5 °C was 3% (8/265). The mean number of inpatient days was 5.7 ± 3.5. There were 29 (10.9%) patients required ICU admission and the mean ICU length of stay was 2.9 ± 1.5 days. Most patients (98.5%, 261/265) recovered completely and 6.9% (4/58) of patients with severe malaria died. There was no association between having parasitaemia 72 h of treatment and response to treatment (P = 0.48). Among 207 patients with uncomplicated malaria, no one died or needed mechanical ventilation and haemodialysis, and one (1%) developed hospital acquired infection. Among 58 patients with severe malaria, eight required haemodialysis, 13 required mechanical ventilation, and 10 developed hospital acquired infection.

Among 29 patients coming from or travelling to Africa, 96.6% (28/29) was male with a median age of 35.1 ± 10.2 years (Table 4). The mean number of days of illness at the time of admission and the mean number of inpatient days were 6.1 ± 5.5 and 5.1 ± 3.8 days, respectively. Three (10.3%) and two (6.9%) patients had fever and parasitaemia 3 days of anti-malarial treatment, respectively. Severe malaria was documented in 8 (27.6%) patients, of whom one developed shock, one developed impaired consciousness, one developed hyperparasitaemia, two developed jaundice, and three developed mixed manifestations. ETF and LTF were recorded in 2 (6.9%) and 8 (27.6%) patients, respectively. Three (10.3%) patients received red blood cell transfusion, two (6.9%) undertook haemodialysis, and three received mechanical ventilation. Two patients died, of whom one patient was 48 years old with atrial fibrillation and type 2 diabetes who admitted to the HTD on day seven of illness with shock and severe metabolic acidosis and another patient was 28 years old who admitted to the HTD on day four of illness with severe metabolic acidosis and impaired consciousness.

**Table 4:** Demographic characteristics, treatment and outcome of 29 *P. falciparum* infected patients from Africa

Characteristics	Summary statistics^a^
Male	96.6 (28)
Age	35.1 ± 10.2
Number of days of illness at the time of admission (days)	6.1 ± 5.5
Number of inpatient days	5.1 ± 3.8
Patients with parasitaemia 72 h of treatment	6.9 (2)
Patients with fever 3 days of treatment	10.3 (3)
Severe malaria	27.6 (8)
Manifestation of severe malaria (n = 8)	
Shock	3.4 (1)
Impaired consciousness	3.4 (1)
Jaundice	6.9 (2)
Hyperparasitaemia	3.4 (1)
Mixed manifestations (i.e., patients had more than one severe manifestation)	10.2 (3)
Early treatment failure	6.9 (2)
Late treatment failure	27.6 (8)
Red blood cell transfusion	10.3 (3)
Haemodialysis	6.9 (2)
Mechanical ventilation	10.3 (3)
Death	6.9 (2)

^a^%(N) for categorical variables and Mean ± SD for continuous variables

### Predictors for severe malaria

Severe malaria was statistically associated with receiving treatment at previous hospitals (P < 0.001), hepatomegaly (P < 0.001) and number of inpatient days (P < 0.001) (Table 5). There were no association between severe malaria and gender, age, BMI, number of days of illness, acquiring malaria before 2017, pregnancy, fever on admission, splenomegaly, and anaemia (P > 0.05).

**Table 5:** Association between patterns of malaria infection and demographic and clinical characteristics

Characteristics^a^	Severe malaria (n = 58)	Uncomplicated malaria (n = 207)	P value*	OR (CI)
Age (years)	37.3 ± 14.2	34.7 ± 12.7	0.17	
Male	82.8 (48)	83.6 (173)	0.9	
BMI	21.5 ± 3.3	21.4 ± 3	0.87	
Number of days of illness	6.8 ± 4.4	6.3 ± 5.2		
Receiving treatment in previous hospital	16 (27.6%)	26 (12.6%)	< 0.001	2.7 (1.3–5.3)
Acquiring malaria before 2017	46.6 (27)	44 (91)	0.72	
Pregnancy	1.7 (1)	0.5 (1)	0.3	
Fever on admission	100 (58)	99.5 (206)	0.6	
Hepatomegaly	31 (18)	4.3 (9)	< 0.001	9.9 (4.2–23.6)
Splenomegaly	5.2 (3)	2.4 (5)	0.3	
Anaemia	1.7 (1)	0.5 (1)	0.3	
Number of inpatient days	7.8 ± 4.2	5 ± 3	< 0.001	

^a^%(N) for categorical variables and Mean ± SD for continuous variables

*Chi-squared test for categorical variables and student t test for continuous variables

### Predictors for early and late treatment failures

Patients with severe malaria were significantly more likely to develop ETF (P < 0.001, OR 7.5, CI 2.8–20.0) and less likely to develop LTF (P = 0.044, OR 0.44, CI 0.19–0.99) compared with those who did not have severe malaria (Table 6). Gender, living in or travelling to malaria endemic areas 14 days prior to the onset of disease, number of days of illness > 7 days at the time of admission, acquiring malaria before 2017 and hyperparasitaemia (P > 0.05) were not a predictor of ETF and LTF. Similarly, having parasitaemia 72 h of treatment and fever 3 days of treatment (P > 0.05) were not predictors of LTF. There is an association between age and ETF (P = 0.027), while there is no association between age and LTF (P = 0.054).

**Table 6:** Unadjusted predictors tested for early and late treatment failures

Predictors	Early treatment failure*				Late treatment failure*			
	Yes (n = 19)	No (n = 246)	P value	OR (95% CI)	Yes (n = 51)	No (n = 214)	P value	OR (95% CI)
Male	84.2 (16)	83.3 (205)	0.5^a^		90.2 (46)	81.8 (175)	0.1^c^	
Living in or travelling to malaria endemic areas 14 days prior to the onset of disease			0.112^b^				0.4^b^	
Living in endemic area	36.8 (7)	45.5 (112)			41.2 (21)	45.8 (98)		
Travel to endemic area	26.3 (5)	37.4 (92)			35.3 (18)	36.9 (79)		
Not living and not traveling to endemic area	36.8 (7)	17.1 (42)			23.5 (12)	17.3 (37)		
Acquiring malaria before 2017	63.2 (12)	43.1 (106)	0.09^c^		47.1 (24)	43.9 (94)	0.7^c^	
Number of days of illness at the time of admission > 7 days	10.5 (2)	26.5 (65)	0.17^a^		29.4 (15)	24.3 (52)	0.5^c^	
Hyperparasitaemia	0	0.4 (1)	1^a^		0	0.5 (1)	1^a^	
Severe malaria	63.2 (12)	18.7 (46)	< 0.001^a^	7.5 (2.8–20.0)	15.7 (8)	29.9 (50)	0.044^c^	0.44 (0.19–0.99)
Age (years)	41.7 ± 12.5	34.8 ± 13.0	0.027^d^		32.1 ± 12.3	36.1 ± 13.2	0.054^d^	
Patients with parasitaemia 72 h of treatment					25 (12)	13.5 (28)	0.047^c^	2.14 (0.99–4.61)
Patients with fever 3 days of treatment					14.6 (6)	15.8 (29)	0.9^c^	

^*^ %(N) for categorical variables and Mean ± SD for continuous variables

^a^ Fisher’s Exact test

^b^ Chi-square for trend test

^c^ Chi-square test

^d^ Student’s t test

### Model for the prediction of early and late treatment failures

No predictor for ETF was identified other than having severe malaria (AOR 6.96, CI 2.55–19.02, P < 0.001) (Table 7). No predictor for LTF was identified.

**Table 7:** Multinominal logistic regression analysis for predictors of early and late treatment failures

Predictors	Early treatment failure		Late treatment failure	
	P	Adjusted OR (95% CI)	P	Adjusted OR (95% CI)
Age (years)	0.062	1.04 (0.998–1.073)	0.11	0.98 (0.95–1.01)
Severe malaria	< 0.001	6.96 (2.55–19.02)	0.094	0.49 (0.21–1.13)
Acquiring malaria before 2017	0.096	2.38 (0.86–6.59)		
Patients with parasitaemia 72 h of treatment			0.11	1.93 (0.87–4.30)

## Discussion

A total of 433 patients with malaria admitted to the HTD between 2015 and 2018. Of these 433 patients more than half (59.8%, CI 55.1−64.4%) acquired *P. falciparum* infection. This rate is lower than the rate of 98% (CI 97.5−98.5%) reported from a provincial hospital in southern Vietnam in 1990 [4, 21]. It is impossible to completely understand the reason of this change due to the differences in the study clinics and referral patterns. However, Goldlust et al. have found that the adoption of ACT may have influenced the decline of malaria including *P. falciparum* in Vietnam [4]. Most patients in the presenting study were male in labour age and more than half (56.3%) of them had jobs related to the forest or worked in areas which are known as malaria endemic areas in Vietnam, Cambodia and Africa [22, 23]. Additionally, more than 80% of patients lived in or travelled to malaria endemic areas within 14 days before the onset of disease which is an established risk for malaria infection [15]. These findings were not surprised since according to Vietnamese culture, breadwinner is perceived as the man’s role in the family, and therefore more men tend to be exposed to malaria infection than women when they work in or travel to malaria endemic areas [24]. At this stage, most malaria morbidities and mortalities occur in 21 out of 58 provinces, of which forested areas of provinces located in Central and Central-Southern Vietnam account for the highest malaria burden in Vietnam [25].

In the presenting study, *P. falciparum* infection was recorded throughout the year and peaked during the months from November to March of the following year. In Vietnam, Lunar New Year holiday which is the most important holiday usually occurs in the second half of this period. It is observed that to financially prepare for the holiday many people travel to malaria endemic areas to work in this period in response to the increase in the number of seasonal jobs in these areas.

Blood transfusion and IDU are considered as malaria risk factors [26, 27]. Only one patient who was suspected to have malaria infection from blood transfusion in the presenting study. Two other cases were injecting drug users who may have shared needles and syringes in the days before their illness developed. Pregnancy is a high risk for developing severe malaria [28]. Fortunately, two pregnant patients in our study had uncomplicated malaria. HIV infection has been reported as an underlying disease that may delays parasite clearance time [29]. However, no-one was found to have HIV infection in the presenting study.

According to the WHO definition of severe falciparum malaria [30] and the Vietnam Ministry of Health guidelines for the management of malaria infection [18], patients were classified into uncomplicated and severe malaria groups. In the presenting study, severe malaria accounted for 21.9% (CI 17.1−27.4%). Also, there was an association between severe malaria and prolonged inpatient days. This is probably due to the prolonged anti-malarial therapy and different severe manifestations which required additional supportive treatment as compared with uncomplicated malaria [31]. Similar to another report [32], an association between hepatomegaly and severe malaria was documented in the presenting study. Severe malaria was also found to be associated with receiving treatment at previous hospitals among study participants. In detail, 62.5% (10/16) of study participants receiving treatment at previous hospitals were incorrectly diagnosed as having dengue haemorrhagic fever or septicaemia. It is well documented that misdiagnosis of malaria at previous hospitals leads to delayed anti-malarial treatment, and thus may facilitate severe malaria [33–35]. In light of this, clinicians should pay more attention on the patient’s epidemiological evidence of malaria to prevent misdiagnosis of this disease.

Regarding the clinical symptoms, similar to previous reports [36, 37], fever was frequent (99.6%), while anaemia was rare (0.8%) in the presenting study. However, splenomegaly was reported to be a common symptom among *P. falciparum* infected patients, while hepatomegaly was not mentioned in another study [37]. In the presenting study, splenomegaly was detected in only 3%, while hepatomegaly was documented in up to 10.2% of study participants. Among 58 cases with severe malaria, the most common manifestations were impaired consciousness (20.7%), jaundice (24.1%) and mixed manifestations (22.4%). Other manifestations of severe malaria included anaemia, renal failure, hyperparasitaemia and shock which are similar to other reports [21, 36]. Although mild elevations of transaminases are common in *Plasmodium* infection, particularly in *P. falciparum* infection, this condition is usually transient [38]. About 30% of study participants had transaminase higher than 40 U/L. Treatment delay of seven days or more is documented to be a risk for severe malaria with multi-organ failure [35]. One-fourth of study population admitted to the HTD after seven days of illness and this included some patients receiving treatment from previous hospitals. In the presenting study, more than one fifth of study participants receiving treatment from previous hospitals had been misdiagnosed as having dengue infection or septicemia. An inappropriate therapy provided in previous hospitals could aggravate patient’s condition [39]. This could explain why some patients who were admitted to the HTD after 7 days of disease developed severe malaria. Therefore, clinicians must be more alerted to malaria infection when patients have a travel history to endemic areas [39]. In addition, severe malaria should be suspected if patients were under inappropriate treatment at previous hospitals [40].

Having a prolonged fever after receiving anti-malarial treatment is considered as a predictor of treatment failure [41, 42]. In addition, prolonged fever indirectly reflects the activity of parasite in the human body and consequently indicates anti-malarial drug resistance [41]. In the presenting study, 13.2% of patients had fever longer than 3 days after using DHA-PPQ which is one of the most effective artemisinin-based combinations [7]. This suggests a reduced susceptibility of DHA-PPQ in our study population. Parasite clearance time is also useful to detect artemisinin resistance [41, 43]. Binh Phuoc province is an endemic area of artemisinin resistance in Vietnam [44]. A study showed that the prevalence of positive blood smear for asexual parasitaemia 72 h after treatment initiation in Binh Phuoc was up to 75% [45]. In our study, nearly 50% of patients coming from Binh Phuoc and 15.1% of patients having parasitaemia 72 h of treatment. However, it was unable to perform molecular tests to detect K13 mutations among these patients to confirm artemisinin resistance due to the nature of a retrospective cohort study.

The rates of ETF and LTF in other areas in Vietnam were found to be not different to those in Binh Phuoc-Central Highland where DHA and PPQ resistance has been confirmed, this raises a big concern about the spread of malaria parasites that are resistant to both DHA and PPQ to other areas in Vietnam. It has been documented that artemisinin resistance has emerged in the GMS, followed by ACT failure, because both artemisinin and partner drug have decreased their susceptibility [45]. According to the most recent study examining the efficacy of DHA-PPQ combination in this region, the prevalence of plasmepsin 2/3 amplification which is a marker of PPQ resistance in Binh Phuoc-Vietnam was 77% and the efficacy of DHA-PPQ on day 42 was only 47.1%, while there was only 7% of cases having fever on the day of recrudescent infection [45]. Given the high prevalence of marker of PPQ resistance, the low efficacy of DHA-PPQ treatment and unclear clinical sign of recrudescent infection, clinically detection of this anti-malarial drug resistance is challenging. In light of this, anti-malarial drug resistance may have spread beyond Binh Phuoc province in Vietnam. Besides, *P. falciparum* infection is prevalent in Africa [1]. The proportion of patients from Africa developing ETF (6.9%) and LTF (27.6%) in the presenting study raises a concern about the risk of artemisinin resistance in Africa. Indeed, although local artemisinin resistance has not been confirmed, unexplained slow parasite clearance times [46] and residual submicroscopic parasitaemia after artemisinin-combination therapy [47] have been reported in Uganda and Kenya, respectively.

DHA-PPQ combination has been widely used as a first-choice therapy for *P. falciparum* infection in many countries including Vietnam [48]. However, the spread of artemisinin and partner drug resistance has caused high treatment failure rates to this combination [49] and subsequently threatens the success of malaria control and elimination [9]. In Vietnam, before 2016, DHA-PPQ treatment dose depended on patient’s age [50]. However, many studies have showed that PPQ under-dosing (< 48 mg/kg) is an important factor for recrudescent parasitaemia [48, 51]. It is documented that the underdose of DHA-PPQ increases recrudescent parasitaemia and sequentially increases treatment failure [48]. Indeed, the underdose of PPQ is a significant predictor for recrudescence with the risk increasing by 13% (95% CI 5.0–21%) for every 5 mg/kg decrease in dose [48]. A pharmacological modelling study has found that compared with DHA-PPQ weight-based doing, age-based dosing increases the risk of underdose of DHA-PPQ, and thus risk of malaria treatment failure and recrudescence [52]. In addition, several studies have found that the DHA-PPQ weigh-based dosing enhances prolonged useful therapeutic life including maximizing the likelihood of rapid clinical and parasitological cure and minimize transmission and retard drug resistance [52–55]. To optimize the effectiveness of DHA-PPQ, the WHO recommends treatment with 3 days of DHA-PPQ weight-based dosing to cover at least two asexual life circles of *P. falciparum* [7]. In September 2016, the Vietnam Ministry of Health adopted the WHO guidelines which have been widely used since 2017 [18]. However, there was no difference in the proportion of ETF and LFT before and after 2017 in the presenting study. There was an increase in the burden of DHA-PPQ resistant parasites in the GMS including Binh Phuoc-Vietnam between 2011 and 2018 [45]. This increased burden of parasites carrying mutations is probably responsible for the unchanged proportions of ETF and LTF before and after 2017 in the presenting study even though the new treatment guidelines have been utilized.

A study on African children with uncomplicated falciparum malaria demonstrated the association between age less than 2 years and delayed parasite clearance [56]. In addition, hyperparasitaemia (> 50,000/ul) was also reported as a predictor of delayed parasite clearance [56]. In the presenting study, there was no association between both two treatment failure types and age and hyperparasitaemia. However, having severe malaria was found to be a significant predictor of ETF (P < 0.001, AOR 6.96, CI 2.55–19.02) which has not been reported elsewhere. In Vietnam, the first-choice treatment of severe falciparum malaria is parenteral artesunate single-therapy for up to 7 days total plus a three-day oral DHA-PPQ combination therapy when the patient can tolerate oral therapy [18]. In the presenting study, only one out of four fatal cases came from Binh Phuoc where artemisinin resistance has been documented and thus, the data are insufficient to reject the current recommendation of using parenteral artesunate as first-line treatment for severe falciparum malaria in Viet Nam. However, the presence of artemisinin resistance which can lead to slow parasite clearance in Southeast Asia including Vietnam [9, 41] threatens the effectiveness of the initial parenteral artesunate single therapy for severe malaria. Indeed, one out of 14 patients with severe malaria coming from regions where artemisinin resistance has been documented had a parasite half-life greater than 6 h. It is probably that the variation in time to receive DHA-PPQ combination therapy and the above-mentioned DHA-PPQ resistance may facilitate ETF among patients with severe malaria. In light of this, to reduce the risk for ETF, robust studies are needed to examine the effectiveness of an early initiation of combination therapy including parenteral artesunate and a parenteral partner drug for severe malaria. It is important to re-evaluate the effectiveness of the current WHO recommended anti-malarial therapy for both uncomplicated and severe malaria as well as to develop new intramuscular or parenteral anti-malarial drugs in the context of anti-malarial drug resistance in the GMS.

This study had some limitations. First, this is a single center study, and thus we may have missed patients with falciparum infection receiving treatment at other hospitals but were not referred to the HTD. However, the HTD is the only major tertiary teaching hospital for infectious diseases including malaria in southern Vietnam and receives not only local patients, but also patients from other countries. This would enhance the generalizability of the study findings. The study interval included the period when the WHO’s new treatment guidelines were adopted. This allowed us to examine the change in the patterns of treatment failure in response to the utilization of the new guidelines. Second, it was unable to perform molecular tests to further examine the magnitude of parasites carrying mutations due to the nature of a retrospective cohort study. Third, examining the presence of parasitaemia on day 28 for all patients was not performed due to the same reason. Some cases who had parasitaemia on day 28 but did not exhibit any clinical symptom may have been missed. Therefore, the burden of LTF documented in this study may be underestimated.

## Conclusions

*Plasmodium falciparum* remains the prevalent malaria parasite in Vietnam. Despite the low mortality rate, severe falciparum malaria is not rare and having severe malaria is a significant predictor of ETF. To reduce the risk for ETF, studies are needed to examine the effectiveness of combination therapy including parenteral artesunate and a parenteral partner drug for severe malaria. The study alerts the risk of the spread of *P. falciparum* that is resistant to both DHA and PPQ to other areas in Vietnam and Africa which are currently known as non-endemic areas of anti-malarial drug resistance. A more comprehensive epidemiological survey using molecular technique in these regions is required to completely understand the magnitude of anti-malarial drug resistance and to design appropriate control strategies.

## Data Availability

The datasets used during the current study are available from the corresponding author on reasonable request.

## Abbreviations

CI: 95% confidence interval
ACT: Artemisinin-based combination therapy
AST and ALT: Aminotransaminases
DHA: Dihydroartemisinin
ETF: Early treatment failure
GMS: Greater Mekong Subregion
HTD: Hospital for Tropical Diseases
IDU: Injecting drug use
PPQ: Piperaquine
WHO: World Health Organization
